# Mental health literacy for social phobia in Ghana: Investigation of gender stereotypes and previous experience for recognition rates and prejudice

**DOI:** 10.1177/00207640231206055

**Published:** 2023-11-16

**Authors:** Peter Adu, Tomas Jurcik, Emmanuel Demah, Patrick T Korang, Dmitry Grigoryev

**Affiliations:** 1 School of Health, Wellington Faculty of Health, Victoria University of Wellington, Wellington, New Zealand; 2 HSE University, Moscow, Russia; 3 Kintampo Health Research Centre, Ghana

**Keywords:** Prejudice, gender stereotypes, ambivalent sexism, social phobia, mental health literacy, Ghana

## Abstract

**Background:**

Mental health literacy (MHL) research has been of substantial interest internationally. Nevertheless, the interplay between beliefs, attitudes, previous experience with mental disorders, and knowledge of Ghanaians on specific mental disorders remains to be understood. The present study explored the interconnectedness between gender stereotypes, prejudice, previous experience with social phobia, and MHL among the general population in Ghana.

**Method:**

Six hundred and one Ghanaians were recruited for an online experimental study design using a survey approach for data gathering. Respondents were randomly assigned to one of the two conditions (i.e., male and female vignettes) depicting symptoms of social phobia for a hypothetical person. Participants further completed self-reported measures including gender stereotypes (based on Ambivalent Sexism Inventory) and prejudice.

**Results:**

Results revealed a 15.5% recognition rate for social phobia. Recognition rates of social phobia did not differ by the experimental condition or by the gender of participants. However, personal experience of social phobia was positively related to an increased likelihood of correctly labeling social phobia among men in the female vignette condition, whereas correct recognition of social phobia was negatively related to prejudice among women in the male vignette condition. In the male vignette condition, men with more hostile sexism attitudes toward men exhibited more prejudice toward their hypothetical male counterpart. In contrast, women with hostile sexism attitudes toward men exhibited less prejudice, but greater benevolent sexism attitudes toward men was associated with more prejudice toward the hypothetical male in the vignette.

**Conclusion:**

Findings from the current study emphasize the role of the cultural milieu in shaping effective mental health interventions. The results also have implications for promoting MHL to reduce prejudice in Ghana and other developing countries in the region.

## Introduction

Mental disorders are common among different populations globally. The World Health Organization (WHO) has estimated that over a third of individuals in most countries meet the criteria for a mental disorder at some point in their lives (Wright et al., 2011; Demyttenaere, 2004; see also World Health Organization [WHO], 2001). However, knowledge regarding mental disorders has been found to relate to better mental health outcomes. For instance, correct recognition of symptoms of mental disorders related positively to early help-seeking for mental disorders (Kitchener & Jorm, 2006; Wright et al., 2007).

The concept of Mental Health Literacy (MHL) as coined by Anthony Francis Jorm et al. in Australia in the mid-1990s conceptualizes MHL as people’s knowledge and beliefs about mental disorders, which can aid in identifying, managing, and possibly preventing mental illnesses. Thus, MHL encompasses four elements: (a) prevention and help-seeking options for mental disorders; (b) first aid strategies needed to help individuals at the onset of mental disorders; (c) effective self-help strategies for mild-to-moderate mental disorders; and (d) one’s ability to identify the development of a specific mental disorder. These components are asserted to aid in the holistic approach to mental health (Jorm et al., 1997). The latter is the focus of the current study, specifically the recognition of social phobia.

The Diagnostic and Statistical Manual of Mental Disorders, fifth edition (DSM–5; American Psychiatric Association [APA], 2013) classifies social phobia as persistent and disproportionate anxiety about specific social situations for fear of being judged negatively, avoidance of social situations that produce the anxiety, and related interference in daily functioning. Among adults, the prevalence rate of social phobia varied between 2 and 5% with female to male ratio of 2.5:1 (Den Boer, 1997). Although there are about 300 disorders listed in the DSM-5, social phobia was chosen for this study as it is found to be associated with significant economic cost in the form of educational underperformance, increased financial dependency, decreased work output, social impairment, and other poor life outcomes (Lipsitz & Schneier, 2000). This disorder is found to have comorbidity with other psychiatric disorders such as depression and alcohol dependence (APA, 2013; Baptista et al., 2011). Despite its debilitating effects, social phobia remains relatively understudied, especially in emerging economies such as Ghana, the context of the current study.

Available community studies have reported low recognition rates for social phobia compared to other mental disorders such as depression and schizophrenia. For instance, In Australia, where most MHL studies were conducted, Reavley and Jorm (2011a) indicated that compared with schizophrenia, PTSD, and depression, social phobia was the least correctly recognized mental disorder (i.e., 9.2%) among 6019 young Australian adults (Attygalle et al., 2017). Jorm et al. (2007) also found that social phobia was often misinterpreted by Australians as shyness or lack of confidence resulting in incorrect labeling of the disorder. In another study, only 3% of participants were found to correctly label social phobia (Reavley & Jorm, 2011b). In London, only a 10.7% correct recognition rate was recorded for social phobia (Furnham & Lousley, 2013). Again, Lynch et al. (2021) reported a 47% correct recognition rate for social phobia as against 67% for depression among adolescents in Ireland. Based on our review, recognition rates regarding social phobia have not yet been investigated within the Ghanaian context. Hence, we also expected less than half of our participants to correctly label social phobia.

Furthermore, many factors have been found to predict recognition rates for mental disorders among different samples globally. For instance, education, age, socioeconomic status, urban residence, and gender are reported to relate to the recognition of different mental disorders (Adu et al., 2021; Attygalle et al., 2017; O’Keeffe et al., 2016). Also, previous experience with mental disorders is found to relate to correct labeling of mental disorders: A study conducted in Australia among 472 rural residents suggested that compared with respondents who reported few or no symptoms of mental problems in the past 12 months, participants with experience of a mental disorder in the same period were more likely to identify more symptoms on the depression scale; and also these individuals opted for professional help for their symptoms (Handley et al., 2018).

Additionally, a UK study that recruited 389 students reported that compared with those without any previous personal experience with mental disorders, individuals with previous personal experience showed significantly higher levels of MHL (Gorczynski et al., 2017). Mendenhall and Frauenholtz (2015) indicated that one predictive factor of MHL among parents of children with mood disorders was a previous experience with mental disorders (see also, Hadjimina & Furnham, 2017). Research exploring personal experience with disorders and MHL is lacking within the Ghanaian context. Consequently, based on the reviewed literature, we hypothesized that previous experience (personal or with a significant other) will positively correlate with recognition of social phobia (H1).

Moreover, gender differences in recognition rates including attitudes toward mental disorders have been observed worldwide in vignette-related studies (Cotton et al., 2006). Studies demonstrated that cultural and gender role disparities impact health outcomes (Travis et al., 2012). In certain societies, women are reviled and at other times revered (Glick & Fiske, 1996). The development of the ambivalent sexism theory comprises a mixture of two complementary sets of gender ideologies or beliefs underlying prescriptive gender stereotypes, namely, hostile sexism (HS) and benevolent sexism (BS). Hostile sexism refers to the negative stereotypes against either women or men. On the other hand, benevolent sexism ideas preach paternalistic and well-intentioned attitudes and behaviors toward men and women (Glick & Fiske, 1996, 2001). For instance, viewing men as providers of women’s needs is an act of benevolence toward men, but this same statement is regarded as hostile toward women as it reduces the woman to an incompetent person who is incapable of meeting her life needs.

An observation of the MHL literature suggests that cultural or traditional ideals exemplified or characterized in gender-stereotypical differences could partially be attributed to the inconclusive findings on attitudes toward individuals with mental disorders and differences in recognition rates for mental disorders (Chong et al., 2016; Hadjimina & Furnham, 2017; Swami, 2012). For example, culture influences the expression of stigma, and idioms of psychological distress embedded in communities render MHL research complex, and dynamic (Berry, 2022; Fonseca-Pedrero, 2018), hence the current findings will shed light on the impact of culturally specific and ambivalent gender roles on MHL, and prejudice.

The cultural characterization of men and women in Ghana is such that as a masculine culture, there is an emphasis on assertiveness, toughness, leadership roles, success, and egoistic attributes for men, implying the expression of benevolent attitudes toward men; women on the other hand, are expected to have the converse of these attributes (Hofstede, 2001), which sometimes indicate hostility toward women. For example, women are expected to care for the home and children and are less favorably considered in high-status leadership positions that demand agentic traits such as independence and self-confidence (Buffington et al., 2016; Clow et al., 2014; Glick & Fiske, 2011). Thus, there is the likelihood of perceiving the behaviors presented in a vulnerable female vignette as typical of women compared to an equally vulnerable male vignette, who instead may be perceived as unusual compared to a “typical Ghanaian man.” Accordingly, consistent with agentic-competency dimensions and the hegemonic masculine nature of Ghanaian society, this study argued that men with hostile beliefs toward men are more likely to exhibit prejudice toward the hypothetical man (H2a), whereas women are more likely to exhibit prejudice toward the hypothetical male when holding benevolent beliefs toward men (H2b).

Relative gender stereotypical differences in MHL evidence shows that both participants’ gender and gender-based vignettes have a significant effect on recognition rates. More specifically, the literature suggests that females have a higher recognition rate for mental disorders compared to males (Hadjimina & Furnham, 2017). Cotton et al. (2006) also found similar results, such that females were more likely to identify the depression vignette correctly compared to males. Males compared with females were better at identifying social phobia (Farrer et al., 2008; Hadjimina & Furnham, 2017). Therefore, we expected that men will be more accurate in identifying social phobia compared to women as more men are educated in Ghana than women (H3). This might go against the pattern found in the West, where men and women are equally educated.

Additionally, in a British representative general population survey study that used gender-based vignettes, Swami (2012) reported significant differences for the gender of the hypothetical persons in the vignette; that is, the study found that men were less likely to indicate that female vignettes suffered from a mental disorder whereas women were more likely to endorse that the male vignette suffered from mental illness. These differences could be attributed to certain culturally based gender expectations in society. Hadjimina and Furnham (2017) found that recognition rates of mental disorders were not associated with vignette gender, although less is known about the influence of vignette gender on the recognition of mental disorders. Hence, researchers expected that participants assigned to the female vignette condition will be more accurate in identifying social phobia than those assigned to the male vignette condition (H4).

Further, recognition rates of mental disorders have been related to mental health prejudice and stigma among various populations worldwide; moreover, such negative attitudes are also found to be associated with poor mental health outcomes (Chekuri et al., 2018; Jang et al., 2012; Vogel et al., 2007). However, literature on MHL and attitudes over the years have concentrated markedly on depression and schizophrenia and prejudice or stigma (e.g., Adu et al., 2021; Angermeyer et al., 2009; Goldney et al., 2005), as perhaps these mental disorders are ranked as key contributors to disability (WHO, 2004, 2017). Less is known about social anxiety, despite high prevalence rates and the potentially disabling nature of this condition (Lipsitz & Schneier, 2000).

Findings available document that knowledge about mental disorders is found to relate to both favorable and unfavorable attitudes toward mentally ill patients. A longitudinal study reported that an increase in knowledge regarding mental disorders, in terms of recognition, help-seeking, and causal beliefs of mental disorders, related positively with mental health prejudice (Angermeyer et al., 2009); however, the reverse was found in New Zealand (Read, 2007). A review reported that in Russia, Germany, Sweden, and Turkey, correct identification of schizophrenia is associated with a higher likelihood of social distancing toward the mentally ill patient (Read, 2007). In one of the few studies on this topic conducted in Ghana, researchers found that recognition of both schizophrenia and depression correlated positively with perceived stigmatizing attitudes in society (Adu et al., 2021, see also; O’Keeffe et al., 2016). The reverse was reported among Singaporeans (Picco et al., 2018) and Chinese (Yin et al., 2020), as correct recognition of mental disorders related negatively to mental health personal and perceived stigma. Such contradictions in the literature could be due to differences in cultural contexts, represented by embodied beliefs and values, thus warranting further research. The extant literature also lacks such attitudinal relations research for social phobia. Although studies on gender and stigma indicated incongruous findings, considering the cultural dimensions of Ghanaian society (see above), we hypothesized that correct recognition of social phobia will relate to unfavorable attitudes toward the male compared to the female vignette condition (H5).

## Method

### Participants

The data for the current study were collected from 601 respondents from the general population in Ghana. That is, 49.4% (*n* = 297) participants were randomly assigned to Condition 1 (male vignettes) and 50.6% (*n*=304) were in Condition 2 (female vignettes), both conditions depicted symptoms of social phobia for a hypothetical person. Participants did not receive any reward for participating in the current study. The sample comprised 66.7% males (*n* = 401) and 33.3% (*n* = 200) females. The age of participants ranged from 18 to 64 years with a mean age of 28.5 (*SD* = 5.4). In terms of education, 54.4% (*n* = 327) of the participants reported holding a Bachelor’s degree, 22.1% (*n* = 133) had a diploma (i.e., 3-year training in either education or nursing), 10.6% (*n* = 64) had a senior high school certificate, 10.3% (*n* = 62) had a Master’s degree, 1.3% (*n* = 8) were Ph.D. holders, 0.3% (*n* = 2) had a basic school certificate, and 0.8% (*n* = 5) did not report their educational attainment. Additionally, 96.5% of participants confirmed that they are Christians, followed by 2.8% Muslims and 0.7% Traditionalists.

#### Power analysis

An a priori power analysis using G*Power 3.1 was conducted to determine the required sample size: an effect size of .03 (*f*^2^), α error probability of .05, and anticipated power of .80 yielded a required sample size of *N* = 531 for these parameters (Faul et al., 2007). The current sample is greater, which contributes to more statistical power.

### Procedure

The current study was approved by the Institutional Review Board (IRB) of the authors (#74). Informed consent was obtained from all individual participants included in the study. We employed an experimental study design utilizing a survey approach for gathering data. The experimental design was employed as the current study manipulated gender to examine its impact on MHL. A single survey link that automatically and randomly assigned participants to either condition (male vs. female vignette) was generated to circulate on different social media platforms (e.g., Facebook, WhatsApp, E-mail, and Twitter). The survey included questions regarding beliefs and attitudes toward this hypothetical person. Participants responded to some basic demographic questions (gender, age, educational qualification, and religious affiliation) before proceeding to the main measures. Participants took about 10 to 15 minutes to complete the online survey. All material was presented in English as it is the official language of Ghana.

Individuals and organizations known to the first author (PA) in Ghana were approached to participate in the anonymous study. Using a snowball sampling approach, these individuals were encouraged to forward the survey link to others they knew. Although online data collection methods present some limitations, we relied on this method for data collection as it is cost-effective and allows for efficient access to broad swaths of the population that are distributed across diverse geographic locations (Lefever et al., 2007). Further, 53% of Ghanaians have access to the internet through various devices (Datareportal, 2022).

### Measures

#### Recognition rates and previous experience

The online Computer-Assisted-Telephone-Interview (CATI) scale (Reavley & Jorm, 2011a) was used to measure MHL (recognition of social phobia) and previous experience with social phobia. This interview scale was modified to an internet-based (SelectSurvey.net) survey. The scale presents a vignette that depicts the social phobia symptoms of a hypothetical person; participants were therefore asked to identify the type of mental disorder depicted in the vignette. This questionnaire was chosen because the vignettes are used in numerous international studies of MHL (e.g., Adu et al., 2021; Jorm et al., 2006; Yoshioka et al., 2014). The name ‘John’ was used in the male condition vignette and ‘Mary’ was used in the female condition vignette: these are common names used in Ghana considered to be ‘tribal free’. The male Social Phobia vignette read as follows: ‘John is a 25-year-old living at home with his parents. Since starting his new university last year, he has become even more shy than usual and has made only one friend . . .’ (see Appendix for the full vignettes). A question followed this scenario: ‘What, if anything, is wrong with the person?’ Participants were required to provide their impressions of the hypothetical person using open-ended responses. Participants further indicated if they or anyone in their social circle had ever experienced such a condition before.

#### Gender stereotypes

The Ambivalent Sexism Inventory (ASI) toward women (Glick & Fiske, 1996) and men (Glick & Fiske, 1999) was used to measure the content of general gender stereotypes in the current survey due to its psychometric properties and popularity in many studies conducted in different countries (Diabah, & Agyepong, 2022; Glick et al., 2016; Rollero et al., 2014). An example of an item on this scale is ‘Many women have a quality of purity that few men possess’. Participants would use a 6-point scale for answering (0 = *strongly disagree*; 5 = *strongly agree*). The internal consistency (Cronbach’s alpha) of all the subscales was considered to be generally acceptable: .71 for benevolence toward women (*M* = 3.27, *SD* = 1.06), .74 for benevolence toward men (*M* = 3.25, *SD* = 1.15), .76 for hostility towards women (*M* = 2.58, *SD* = 1.16), and .68 for hostility toward men (*M* = 2.76, *SD* = 1.09).

#### Prejudice

The feeling thermometer scale was used to measure attitudes toward the vignette with social phobia. This popular scale provides participants the opportunity to indicate on a scale (feeling thermometer) from 0 to 100 how they feel toward the hypothetical person (i.e., the scale is in three parts: 0–50 is a cold feeling, 50 is a neutral feeling, and 50–100 is warm feeling). We reverse coded this scale for the analysis so that the maximum value of the scale was equal to the extreme expression of prejudice (i.e., from 0 = *warm feeling* to 100 = *cold feeling; M* = 52.33, *SD* = 24.12). These instruments were combined to form a single questionnaire in the current study. The data used for the current paper is part of a larger project on MHL in Ghana.

### Data analysis

Statistical Package for Social Sciences (SPSS) version 27 was used to analyze the data. The data from the online questionnaire was exported from the online survey software to the SPSS software. The data were screened for missing values and inspected using Little’s MCAR test (Little, 1988). The data were not missing completely at random (*p* > .05), hence, the Expectation Maximization (EM) algorithm was computed to cope with a few missing values (Dempster et al., 1977). The normal distribution assumption was met as kurtosis and skewness were within the range of −3 to 3, hence it was acceptable to use parametric statistics (Tabachnick & Fidell, 2018).

Descriptive statistics were computed for demographic variables and subscale scores. This was followed by a reliability analysis for all non-binary scales/subscales. Descriptive statistics were used to test hypothesis one; hypotheses four and five were tested using the chi-square test; hypothesis two was tested with logistic regression; and finally, hypotheses three and six were tested with multiple linear regression.

## Results

### Recognition rates

Concerning respondents’ recognition and exposure to social phobia, evidence showed that out of 601 participants, only 15.5% correctly identified social phobia (Table 1). Results further indicate that the majority of the participants (51.6%) do not have any exposure to others with a social phobia as compared to 47% who confirmed their personal experience with social phobia. In a similar vein, results showed that there was no significant gender difference in recognition of social phobia among the sample χ^2^(1) = 0.32, *p* = .575. Relatedly, no significant difference in recognition of social phobia among the two conditions was observed χ^2^(1) = 0.31, *p* = .577.

**Table 1.** Proportion of social phobia recognition by gender and vignette conditions (*N* = 601).

	Social phobia recognition	
	Incorrect identification	Correct identification
Gender		
Man	341	59
Woman	167	33
Condition		
Male vignette	259	44
Female vignette	249	48

*Note.* Indices for social phobia recognition by gender [χ^2^(1) = 0.315, *p* = .575] and social phobia recognition by gender-based vignette conditions [χ^2^(1) = 0.311, *p* = .577].

### Recognition rate, previous experience with social phobia, and gender stereotypes

Table 2 revealed that men who were self-exposed to mental health disorders were more likely to recognize social phobia in the female condition (*OR* = 4.813, *p* = .002). On the other hand, there was no evidence that exposure to others with social phobia significantly predicted recognition of social phobia among the participants. Moreover, no evidence that ambivalent sexism for men and women in both conditions predicted recognition of social phobia.

**Table 2.** Logistic regression for recognition of social phobia (*N* = 601).

	Men		Women	
	Female vignette	Male vignette	Female vignette	Male vignette
Sociodemographic variables				
Age	0.929	1.037	0.952	0.880
Education	1.533	1.068	1.065	1.435
Exposure				
Self (1 = yes) H2	4.813**	0.668	1.105	0.823
Others (1 = yes)	0.269	0.684	0.856	0.967
Ambivalent sexism				
Benevolence toward women	0.972		0.742	
Hostility toward women	1.203		0.931	
Benevolence toward men		1.312		0.560
Hostility toward men		1.076		1.267
*R*^2^	.10	.03	.03	.05

** *p* < .01.

### Gender stereotypes and prejudice

In the linear regression model, the current study tested factors that predict prejudice toward the person with social phobia. Among them include sociodemographic variables, exposure to social phobia, and ambivalent sexism. Results from Table 3 show that age positively predicted prejudice among women in the male condition (β = .264, *p* = .009) while among women participants, education negatively predicted prejudice in the male condition (β = −.250, *p* = .012). Moreover, women participants who reported personal exposure to social phobia were less likely to be prejudiced in both male and female conditions (β = −.314, *p* = .006; β = −.269, *p* = .010, respectively). The same pattern was found for men, but it failed to reach significance. Interestingly, our results revealed that women with exposure to others with social phobia were more likely to be prejudiced in the male condition (β = .354, *p* < .002). However, within the male condition, women who correctly recognized social phobia were less likely to exhibit prejudice (β = .200, *p* = .036).

**Table 3.** Linear regression for prejudice (*N* = 601).

	Men		Women	
	Female vignette	Male vignette	Female vignette	Male vignette
Sociodemographic variables				
Age	−.015	.137	−.018	.264**
Education	−.058	−.079	−.058	−.250*
Exposure				
Self (1 = yes)	−.137	−.134	−.269**	−.314**
Others (1 = yes)	.061	−.058	.162	.354**
Recognition (1 = yes)	−.037	−.094	−.118	−.200*
Ambivalent sexism				
Benevolence toward women	.172		.167	
Hostility toward women	−.179		−.082	
Benevolence toward men		−.122		.256*
Hostility toward men		.168*		−.243*
*R*^2^	.05	.06	.11	.30

*p < .05. **p < .01.

Further results showed that women who held benevolent attitudes toward men were more likely to report prejudice (β = .256, *p* = .020) in the male condition, while women who held hostile attitudes toward men were less likely to report prejudice (β = −.243, *p* = .024). Meanwhile, men who held hostile attitudes toward men are more likely to be prejudiced in the male condition (β = .168, *p* = .049).

### Discussion

The current study sought to experimentally explore the interconnection between Mental Health Literacy (MHL), gender stereotypes, previous experience with mental disorders, and prejudice regarding social phobia. In total, five hypotheses underlined the study of which one was fully supported, one was partially supported, one revealed counter findings, and two were not supported by the data available. That is, respectively, men with hostile beliefs toward men exhibited prejudice toward the hypothetical person in the male condition (H2a), women with benevolent beliefs toward men showed prejudice toward the hypothetical person in the male condition (H2b), personal previous experience of social phobia related positively with recognition of social phobia (H1). Notwithstanding, there was no gender difference in the recognition rate regarding social phobia (H3), there was no difference between the two conditions (male vs. female vignette) in the recognition of social phobia (H4), and finally, counter to what was expected, correct recognition of social phobia among women in the male vignette related negatively with prejudice toward the hypothetical male (H5). It is worth noting that there was a relatively low recognition rate of social phobia among participants, that is, as we anticipated less than half of the participants correctly recognized social phobia.

### Recognition rates

The evidence available has reported low recognition rates for social phobia in the literature. In tandem with other studies, the current research supported such assertions. That is, the substantial proportion of the participants who under-recognized social phobia in the current study makes our findings congruous with those obtained in a study conducted in Australia and Sri Lanka (Attygalle et al., 2017; Reavley & Jorm, 2011a). These studies reported that as few as 3% of Australian respondents were able to recognize social phobia (Reavley & Jorm, 2011b). Notably, there were differences in the samples used in these studies. For example, participants used in the current study were predominately educated; in London, participants were undergraduate psychology students (Furnham & Lousley, 2013); and adolescents were used in the Sir Lankan study (Attygalle et al., 2017). Importantly, the under-recognition of social phobia in these varying samples shows that MHL for social phobia is low within the general population worldwide; social phobia is also less studied compared to depression and schizophrenia (Adu et al., 2021; US Department of Health and Human Services, 2010).

There was no gender difference in the recognition rate for social phobia, and we did not observe differences between the two gendered vignette conditions in terms of recognition rates. Thus, both genders plausibly exhibited equivalent knowledge of social phobia. Gender differences have emerged elsewhere (e.g., Furnham & Lousley, 2013), hence the need to assess recognition abilities in different samples worldwide to better tailor MHL interventions. Also, it could be that greater power (sample size) may have allowed us to detect such differences.

### Recognition rate and prejudice

Further, we hypothesized a positive relationship between recognition rate and prejudice toward the male vignette; however, our data revealed the reverse. Thus, our results showed that recognition of social phobia correlated negatively with prejudice toward the male hypothetical person among women assigned to the male condition. Maybe these women possess corrective information regarding social phobia which is related to favorable attitudes toward the hypothetical man in the vignette. Alternatively, from a Ghanaian cultural perspective, women serve as ‘mothers’ for men even in marriages (e.g., feeding men), and as a result, these women participants may have embodied a motherly, accepting role toward this hypothetical and vulnerable male (Glick & Fiske, 1996).

There are mixed findings regarding MHL and prejudice regarding certain mental disorders such as depression, and schizophrenia; some studies reported a negative relation between MHL and prejudice while others reported the reverse (Angermeyer et al., 2009; Read, 2007). Our study extends the literature by providing evidence regarding one of the least studied mental disorders (i.e., social phobia) in recognition and prejudice. Our finding conforms with MHL studies conducted among Singaporeans (Picco et al., 2018) and Chinese (Yin et al., 2020) as correct recognition of mental disorders related negatively to mental health prejudice. Of note, the mental disorders used in the aforementioned studies differed from that of the current study. Saliently, and contrary to the literature, a study conducted in Ghana reported a positive relation between mental health stigma and recognition of depression and schizophrenia (Adu et al., 2021). Such differences in the same context could result from disparities in the type of mental disorder vignettes depicted in these studies, and in the way stigma was assessed. Thus, prejudice reduction interventions may be adapted to specific attitudes toward each mental disorder.

### Recognition rate and previous experience of mental disorder

In concordance with previous literature, our study found a positive association between recognition of social phobia and previous experience of social phobia. In the UK, students with a personal history of mental disorders showed significantly higher levels of MHL (Gorczynski et al., 2017; see also, Handley et al., 2018; Hadjimina & Furnham, 2017). Despite the differences between these two studies in terms of samples and measures of MHL, it is said that ‘sometimes experience is the best teacher’. Indeed, this adage applied to our findings implies that individuals who personally experienced social phobia sometime in their lives tended to be familiar with the symptoms of the disorder, and consequently were able to correctly label it. Unexpectedly, the experience of others with social phobia did not predict the recognition rate in the current study, suggesting that personal experience may be more salient.

### Prejudice and gender stereotypes

Intriguingly, our results showed that men with hostile attitudes toward men exhibited prejudice toward the male vignette with social anxiety but not the female hypothetical person; this finding could be elucidated from the perspective of hegemonic masculinity in Ghanaian society. A ‘typical Ghanaian man’ is expected to be tough, strong, and bold (Hofstede, 2001), hence the likelihood to perceive the hypothetical male characteristics as unfavorable or atypical of this culturally defined Ghanaian man. And since this ideology is pronounced among both genders, it is perhaps not surprising that men exhibited unfavorable attitudes toward this hypothetical counterpart in the male condition (Dery & Akurugu, 2021), and women with benevolent attitudes toward men showed more prejudice toward the hypothetical male with social phobia. Perhaps women with benevolent attitudes continuously endorse the Ghanaian ideology of a typical Ghanaian man, as a result, these women could perceive the exhibited behaviors depicted in the vignette as unfavorable. Although possessing benevolent attitudes toward others communicates an act of kindness and acceptance, as well as favorable attitudes toward others (Buetow, 2013; Glick & Fiske, 1996), women with hostile sexism attitudes paradoxically showed less prejudice toward the male hypothetical person. This finding implies that women who are hostile to male gender roles may be more accepting of men who do not follow such roles and show vulnerability. Our results suggest the influence of culture on mental health-related attitudes (Clark et al., 2020)

### Strength and limitations

A few limitations need to be acknowledged in the current study. Survey study designs frequently utilize samples from the population rather than encompassing the entire population, as was the case with our study’s sample. Unfortunately, our sample was not fully representative of the Ghanaian population, which subsequently limits the generalizability of our results. As the study used an online data collection method, most of the participants were educated, and more than half of the sample had completed graduate education. Over 60% of the sample were men, indicating a gender bias among the sample. These demographics further highlight the predominance of educated participants as more men are educated than women in Ghana. Moreso, the use of vignettes in MHL research has been criticized in the literature as diagnostic vignettes fail to capture all components of MHL, and evidence regarding the use of vignettes to improve MHL is limited as it becomes difficult to differentiate between simple symptoms of disorders and full syndromes and daily experience of distress (Pescosolido et al., 2008; see a review, Kutcher et al., 2016). Finally, the reliance on self-report measures in our survey methodology could potentially introduce bias into the results, particularly when administered in a single assessment session. That is, participants may give socially desirable answers, choose a pattern for their response, or make guesses that artifictually inflate a potential correlation; hence, results obtained in the current study should be interpreted with caution (Podsakoff et al., 2003). Vignettes may provide low ecological validity as participants may behave differently in real situations compared to hypothetical ones.

Nonetheless, to the best of our knowledge, the current study provides important novelty in MHL regarding the interconnections between the identification of social phobia, gender stereotypes, previous experience of mental disorders, and prejudice among a Ghanaian sample. Thus, our study is the first to explore the relation between the aforementioned variables in the literature, and has therefore the potential to theoretically contribute to a better understanding and conceptualization of social phobia as it relates to gender stereotypes in the Ghanaian context. Practically, our study has will hopefully inspire future replications in a more representative sample, and support the incusion of certain gender and cultural elements in the MHL literature.

### Implication and future directions

Theoretically, the low recognition rates of social phobia across diverse cultures point to the importance of channeling MHL campaigns in this direction. Using more culturally specific standardized vignettes may help promote more reliable results, given that mental disorders are expressed differently across cultural contexts (Heine, 2015; Jurcik et al., 2013; Thombs et al., 2008). Such standardization of vignettes may be preceded by an investigation of cultural conceptualization and formulations of mental disorders. Cultural psychiatry has highlighted the importance of the ‘idiom of expression’ (i.e., how cultural groups express psychological distress) in the diagnoses, treatment, and implementation of mental health interventions (see also Berry, 2022). Our results on the correlation between gender stereotypes and mental health prejudice imply that campaigns intended to address prejudices in societies should consider cultural elements in their campaign strategies that also may be tailored to groups of individuals who endorse or reject particular gender roles. We also believe that large cross-cultural projects such as ManyLabs (see e.g., Van Bavel et al., 2022) would benefit from integrating the field of MHL into their work.

Future research may consider using a more representative and gender balance sample to explore the relation between these variables to provide more insight regarding how different mental disorders are perceived, or how culturally defined gender roles impact MHL. Future studies may benefit from investigating these variables in a real-life situation as opposed to a hypothetical situation. A longitudinal examination of these variables could be advantageous to mental health policies, and incorporating other indicators of MHL (e.g., help seeking) in future studies may help provide a more holistic view of MHL regarding social phobia within the Ghanaian context.

### Conclusion

Our study examined the association between an understudied mental disorder (social phobia) in the MHL field, gender stereotypes, the experience of mental disorders, and prejudice using an experimental survey design. Researchers showed that overall, recognition of social phobia was relatively low among participants, gender differences in recognition rate were not significant, and the two experimental groups (male vs. female vignette) did not differ in their identification of social phobia. Further, results revealed that having personally experienced social phobia related to an increased likelihood of correctly labeling social phobia, being able to correctly label social phobia was linked to prejudice toward the male gender condition. Unsurprisingly, men with more hostile attitudes toward men exhibited more prejudice toward their hypothetical male counterpart, and women with benevolent attitudes showed more prejudice toward the hypothetical male vignette.

While our study addresses an important gap in the MHL literature on social phobia, it also highlights the vitality of the impact of gendered attitudes in the Ghanaian cultural context on health-related behaviors. More research using representative samples addressing casual relations is required for more confident conclusions in this regard. The question remains whether results would be consistent in different sub-samples within the Ghanaian context considering the agentic-competency dimensions and hegemonic masculine cultural aspects of this society.

## Appendix

### Male vignette

John is a 25-year-old living at home with his parents. Since starting his new University last year, he has become even more shy than usual and has made only one friend. He would really like to make more friends but is scared that he’ll do or say something embarrassing when he’s around others. Although John’s work is OK he rarely says a word in class and becomes incredibly nervous, trembles, blushes and seems like he might vomit if he has to answer a question or speak in front of the class. At home, John is quite talkative with his family, but becomes quiet if anyone he doesn’t know well comes over. He never answers the phone, and he refuses to attend social gatherings. He knows his fears are unreasonable, but he can’t seem to control them, and this really upsets him.

### Female vignette

Mary is a 25-year old living at home with her parents. Since starting her new University last year she has become even more shy than usual and has made only one friend. She would really like to make more friends but is scared that she’ll do or say something embarrassing when she’s around others. Although Mary’s work is OK she rarely says a word in class and becomes incredibly nervous, trembles, blushes, and seems like she might vomit if she has to answer a question or speak in front of the class. At home, Mary is quite talkative with her family, but becomes quiet if anyone she doesn’t know well comes over. She never answers the phone and she refuses to attend social gatherings. She knows her fears are unreasonable but she can’t seem to control them, and this really upsets her.
